# Coating and Patterning Functional Materials for Large Area Electrofluidic Arrays

**DOI:** 10.3390/ma9080707

**Published:** 2016-08-19

**Authors:** Hao Wu, Biao Tang, Robert A. Hayes, Yingying Dou, Yuanyuan Guo, Hongwei Jiang, Guofu Zhou

**Affiliations:** 1 Electronic Paper Display Institute, South China Academy of Advanced Optoelectronics, South China Normal University, Higher Education Mega Center, Guangzhou 510006, China; wuhao@scnu.edu.cn (H.W.); douyingying@scnu.edu.cn (Y.D.); yuanyuan.guo@guohua-oet.com (Y.G.); hongwei.jiang@guohua-oet.com (H.J.); guofu.zhou@m.scnu.edu.cn (G.Z.); 2 Shenzhen Guohua Optoelectronics Tech. Co. Ltd., Shenzhen 518110, China

**Keywords:** electrofluidic, functional materials, large area arrays, printing technology

## Abstract

Industrialization of electrofluidic devices requires both high performance coating laminates and efficient material utilization on large area substrates. Here we show that screen printing can be effectively used to provide homogeneous pin-hole free patterned amorphous fluoropolymer dielectric layers to provide both the insulating and fluidic reversibility required for devices. Subsequently, we over-coat photoresist using slit coating on this normally extremely hydrophobic layer. In this way, we are able to pattern the photoresist by conventional lithography to provide the chemical contrast required for liquids dosing by self-assembly and highly-reversible electrofluidic switching. Materials, interfacial chemistry, and processing all contribute to the provision of the required engineered substrate properties. Coating homogeneity as characterized by metrology and device performance data are used to validate the methodology, which is well-suited for transfer to high volume production in existing LCD cell-making facilities.

## 1. Introduction

Electrofluidics, also known as electrowetting, is a mechanism of changing the wettability of a dielectric solid surface by use of an applied electric field. It has been widely investigated as a tool for the manipulation of liquids at miniature scales [1,2], with applications including displays [3,4,5], lab-on-a-chip [6,7,8], and optic lenses [9,10,11]. The commercial feasibility of electrowetting has been proven, an example being the Parrot Varioptic Arctic “liquid lens” product, which provides a large optical range (Arctic 39N0, for instance, is from −5 to +15 diopters) for a 3~4 mm diameter aperture with a response time of only tens of milliseconds. Apart from devices like the liquid lens [9,10,11] which require a small-size 3-dimensional electrowetting structure within a single cell, there are many applications that require large-scale electrofluidic arrays to achieve their function, such as electrofluidic displays (EFD) [3,4,5], smart windows [12,13], optical valves (for X-ray, UV, IR), and lab-on-chip [14]. However, despite a lot of activities [15], there have as yet been no successfully industrialized electrofluidic array-based concepts.

One of the major challenges is to develop an efficient approach to processing the main functional materials and achieving high quality large area electrofluidic arrays. A number of researchers have reported the fabrication of complex cell structures on small-scale samples [16,17,18]. Some key issues that are important for the commercialization of electrofluidic arrays are simplifying the fabrication process, improving material utilization, and increasing the homogeneity of the coatings over large areas. In our previous work [19,20], we reported the possibility of using screen printing to simultaneously coat and pattern fluoropolymer (FP) films as insulators for EFD devices on 6 inch square substrates. This reduced the coating time from ~4 min/plate to less than 1 min/plate while increasing the material utilization from 22% to >52%. Here we show that electrofluidic arrays can be successfully fabricated on much larger 400 mm × 500 mm substrates using a pilot manufacturing line with key equipment including a screen printer for FP processing and a slit coater for photoresist (PR) coating. The material utilization and process speed have been significantly improved. To validate the methodology and the quality of the engineered substrates, we incorporate the electrofluidic arrays into EFD devices. Display devices are assembled and test data obtained for the purposes of process validation.

## 2. Results and Discussion

### 2.1. Screen Printing of the Amorphous Fluoropolymer

Screen printing is well established in the solar cell industry [21,22] and for organic light-emitting devices [23] yielding of high-quality pin-hole free coatings over large areas. In the present work, the screen mesh count was 200, meaning that there are 200 fibre strands per inch. The diameter of the fibers was 48 μm, and the mesh was arranged at a 45° angle to the process direction. Photosensitive emulsion with a thickness of 15 μm was applied to define the pattern to be printed; the fluoropolymer can only be deposited through the image area. Moreover, to ensure that the thickness is as homogeneous as possible over the entire plate, the size of the frame was about two times larger than the coated substrate, with a size of 900 mm × 900 mm.

**Figure 1:**
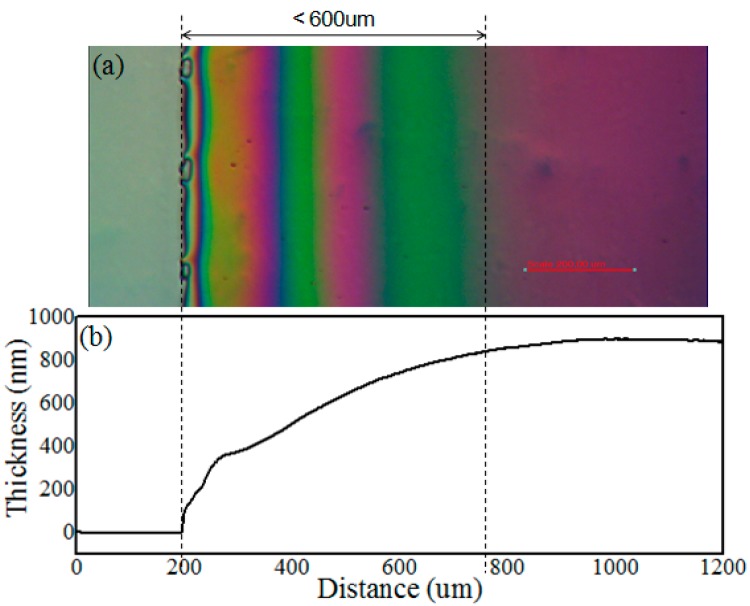
(**a**) Optical micrograph and (**b**) surface morphology data measured by stylus profiler at the edge of the fluoropolymer pad.

To meet the rheological requirements for screen printing and the thickness requirement to provide adequate insulation, formulation of 2–5 wt % AF1600 (Chemours) dissolved in FC-43 or an alternative perfluorinated solvent with similar boiling point was carried out. After the screen printing process, we obtained a relatively homogeneous coating over the entire printed area, as shown in Figure 1. Twelve points are typically tested (Figure S1) on each 400 mm × 500 mm substrate, obtaining an average thickness of ~0.84 μm with average deviation of 2.5%. The uniformity of the coating layer thickness (*d*) has a direct bearing on the electrofluidic response to applied voltage ($V\propto \sqrt{d}$). The uniformity of the dielectric layer can be further improved by optimizing the mesh design parameters, the coating fluid properties, and the printing process. As observed previously [19], the main parameters that affect the thickness of the film are the free mesh volume and the polymer concentration in the coating solution, rather than the printing pressure and speed. We found that in terms of the “pin-hole free” property of the coating, the FP coating processed by screen printing was even better than that prepared by the traditional spin-coating method. Electrical testing, including capacitance and dielectric loss factor measurements are presented in Section 3.1.

Compared with the normal fluoropolymer coating methods, such as dip or spin coating, screen printing is much faster. A 400 mm × 500 mm substrate can be coated in 5 s with a coating speed of 100 mm/s. An additional advantage of screen printing allows direct patterning of the fluoropolymer, which is otherwise carried out by reactive ion etching (RIE) treatment, resulting in further improvement in process efficiency.

Figure 1a shows the colored interference pattern due to variation in thickness in the neighbourhood of the fluoropolymer border. From the microscope image (Figure 1a), we can see that the length of the “rainbow” area corresponding to thickness inhomogeneity extends for 600 μm. The thickness data obtained by stylus profiler coincides with the optical microscope observation, as shown in Figure 1b. Beyond 600 μm from the edge, the thickness variation falls well within the long-range variation in the fluoropolymer thickness over pad and plate level. There is no obvious influence on device performance resulting from the fluctuation of the film thickness away from the pad edge. To accommodate this variation, when designing the pad size, we ensure that it extends beyond the active device edge by 1 mm.

Aside from the process inefficiency, another drawback of spin or dip coating is low material utilization. Here we calculate the utilization (*U*) by the equations below:

(1) $$U=\frac{m_{calc}}{m_{actual}}$$

(2) $$m_{calc}=dA\rho_{FP}$$

(3) $$m_{actual}=m_{added}-m_{coll}$$

where *m_calc_* is the solution weight required for a dry coating thickness, *d* is the thickness of the film, A is the area of the FP, $\rho_{FP}$ is the density of the FP, *m_added_* is the weight of FP solution poured onto the screen, and *m_coll_* is the weight of solution collected from screen after batch completion.

**Figure 2:**
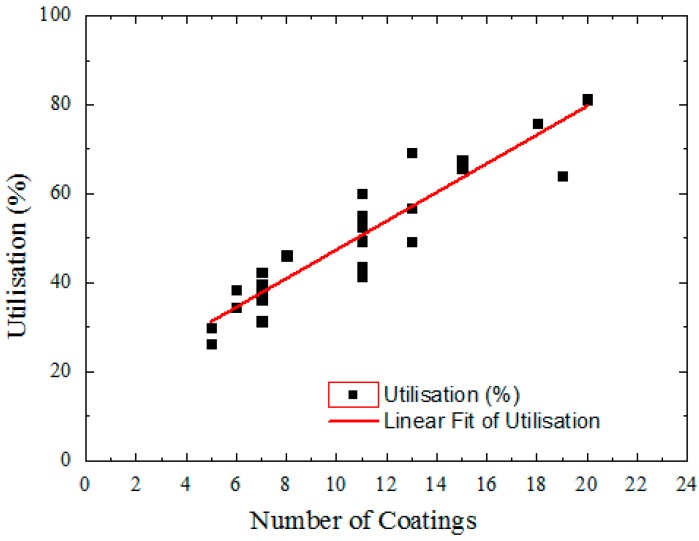
Amorphous fluoropolymer utilization depending on number of coatings printed.

Based on our experience, in excess of 2 g of solution is required to spin coat a 6 inch square plate with a thickness of ~0.8 μm. For an active area of 50% of the plate, the material utilization efficiency will be 22%. In contrast, screen printing has intrinsically high material utilization efficiency. Figure 2 shows the utilization of fluoropolymer solution by the screen printing process, depending on the number of consecutive coatings. The utilization of materials increased with the number of coatings. We have achieved more than 80% material utilization when coating layers of fluoropolymer onto indium tin oxide (ITO) glass (Figure 2). In continuous production, this utilization can be expected to increase further. It is clear that material utilization for screen printing is much higher than other typical coating methods. At the same time, there is still material being left on the screen at completion of the process, which cannot be collected. The utilization of materials has been improved by introducing a standard recycling process.

### 2.2. Slit-Coating Photoresist and Lithographic Patterning

Photoresist arrays were fabricated on the hydrophobic dielectric layer by slit-coating and a single step lithography process. Before coating photoresist, the wettability of the fluoropolymer surface was modified by reactive ion etching. The hydrophobic surface with a receding water contact angle of 100° was made more hydrophilic (receding water contact angle of ~50°), which enables the photoresist to be slit-coated. A typical slit-coating speed is 25 mm/s, therefore taking 20 s per 500 mm plate. An example of the thickness uniformity of photoresist layers (Figure S2) shows the average thickness to be 6.90 μm with an average deviation of 2%.

**Figure 3:**
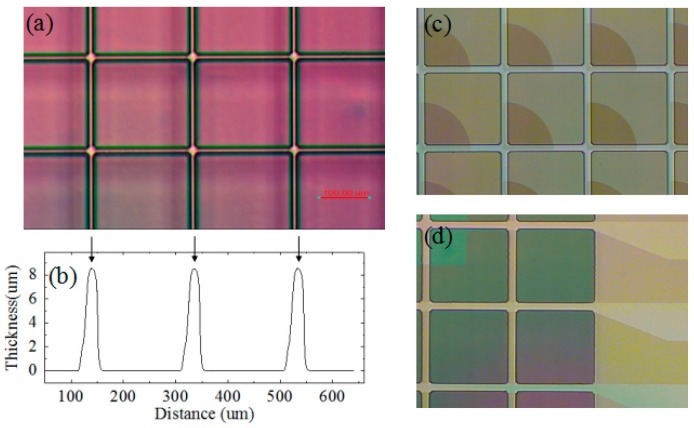
(**a**) Optical microscope photograph and (**b**) morphology data measured by stylus profiler of pixels as an example of the photoresist pattern; (**c**,**d**) Examples of patterning photoresist onto fluoropolymer surface with substrates of various indium tin oxide (ITO) designs.

Production lithography equipment, including exposure equipment with auto alignment function, hot plate line, and developing line were used to pattern negative type photoresist co-developed with a local material supplier and for fabricating array structures. Figure 3a shows an example of the photoresist pattern. The thickness data was obtained by stylus profiler (Figure 3b). In this case, the pixel size is 200 μm × 200 μm with a wall of 10 μm in width and 8 μm in height. Different patterns can be achieved by changing mask design. Figure 3c,d illustrate the lithography on patterned ITO glass coated with a layer of FP film. The pixel structure can be aligned on the underlying ITO pattern with high-precision. The ITO pattern can be in any design, based on the desired purpose. For these two cases (Figure 3c,d), the cell size was also 200 μm × 200 μm with a 15 μm wide wall. With the combination of screen-printing and slit-coating process, large area electric-fluidic structures can be fabricated onto various ITO and TFT (thin film transistor) substrates. Moreover, flexible substrates are also a good option, based on the fluidic fundamentals of electrofluidic devices. In this case, low-temperature processing would be advantageous.

**Figure 4:**
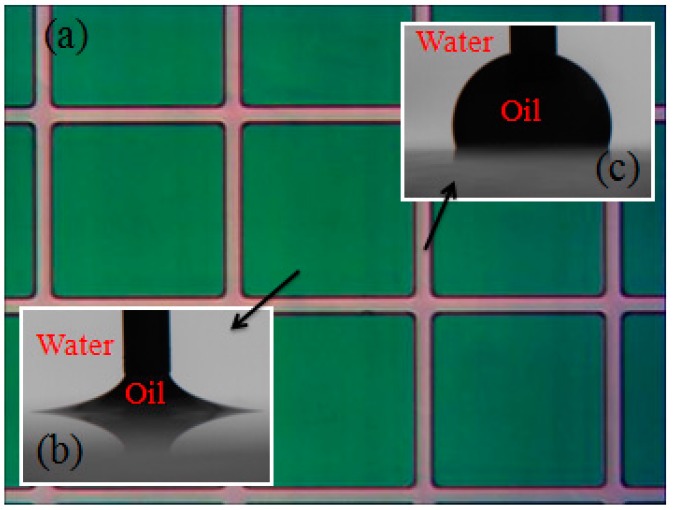
(**a**) Patterned electrofluidic arrays and (**b**) corresponding receding water contact angles of FP and (**c**) photoresist pixel wall materials. The syringe needle for oil dosing is also pictured.

After a thermal anneal process at a temperature of 200 °C for 2 h (>*T*g for FP), we obtained electrofluidic arrays with a hydrophobic FP surface within the cell and a relatively hydrophilic wall. Figure 4 shows the water contact angle of the surface in the cell and the barrier wall. The receding water/oil contact angle of the FP surface in the cell exceeded 170°, while it was 74° on the photoresist wall. We note that although the contact angle hysteresis is extremely low on the FP, the contact angle shows significant hysteresis with strong pinning of the contact line on the crystalline photoresist wall material.

## 3. Device Performance

**Figure 5:**
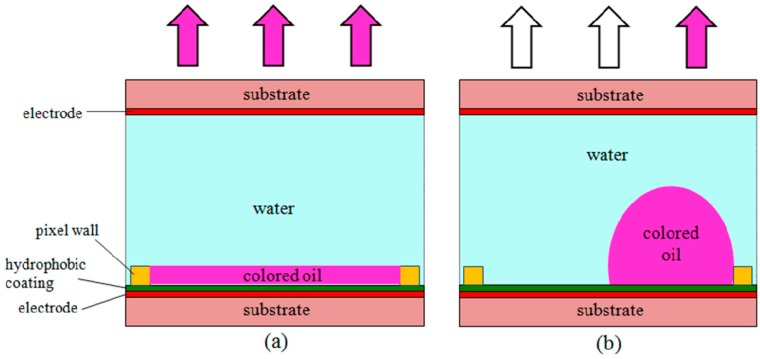
Schematic illustration of the electrofluidic display (EFD) principle. (**a**) In an EFD pixel, without applied voltage, a homogeneous oil film spreads over the pixel area, exhibiting the color of the dyed oil; (**b**) Under applied voltage, the oil film contracts to a corner of the pixel, exposing the color of the underlying substrate.

To demonstrate the practicability and utility of these electrofluidic arrays, we used a typical application—displays. Electrowetting-based display pixels were first reported in 2003 by Hayes and Feenstra [3]. This aroused the attention of many researchers [4,5,24,25]. The basic mechanism and individual pixel structure is shown in Figure 5. Polar liquid (such as aqueous solution) and colored oil form a stack on the surface of the hydrophobic insulator layer-covered electrode substrate. When there is no voltage, interfacial tension forces between the water/oil/dielectric result in a water contact angle exceeding 170°. An oil film is formed. Therefore, the oil (which has a very small complementary contact angle of <10°) spreads across the surface and provides coloration [26]. Applying voltage to this system causes the water to wet the hydrophobic dielectric—the oil converging to a corner of the pixel with the surface coloration altered.

In EFD devices, the electrofluidic array substrates were utilized as engineered substrate. The “engineered substrate” refers to the substrate before liquids dosing, and comprises substrate, electrode, hydrophobic coating, and pixel wall. Engineered substrates can provide numerous pixel structures; for example, there are ~360,000 pixels in a 3-inch square screen with a resolution of 200 ppi (pixels per inch). Manufacturing uniform electrofluidic arrays is critical, because a thickness variation of hydrophobic insulator layer [2,18,27], defects in the insulator coating [27], height changes of pixel, or a delamination of the layers will all cause problems with device performance and reliability.

### 3.1. Electrical Characterization of EFD Cells

**Figure 6:**
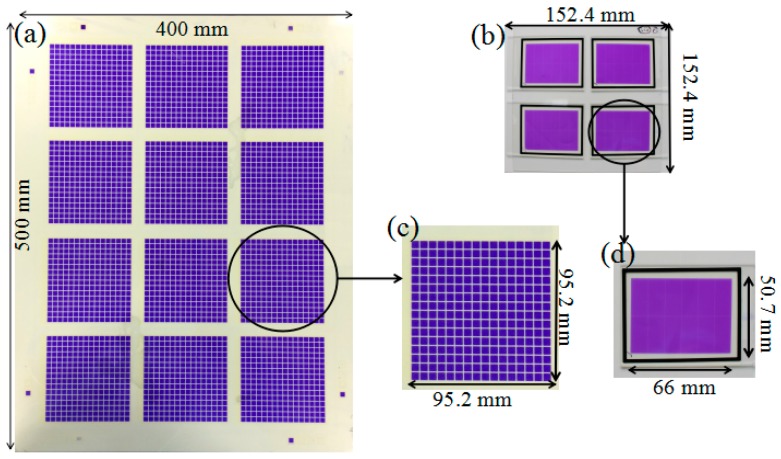
(**a**) A standard 400 mm × 500 mm-sized EFD sample fabricated with an engineered substrate processed by printing; (**b**) A standard 6 inch (152.4 mm × 152.4 mm) sample fabricated by a spin coating process; (**c**) A “cell” unit of sample (a) of size 92.5 mm × 92.5 mm; (**d**) A “cell” unit of sample (b) of size 50.7 mm × 66 mm.

The EFD screens were assembled by filling water and oil into the electrofluidic array substrates and coupling the cover plate with ground electrode for water. The filling process essentially involves self assembly as described in [4], the quality of which is facilitated by the chemical contrast provided by a properly engineered substrate. An example is shown in Figure 6a. Figure 6c is the “cell” unit with size of 95.2 × 95.2 mm^2^ forming part of a 4 × 3 array on the 400 mm × 500 mm sized “mother glass”. To characterize the pin-hole free property of the FP films and the homogeneity of both FP coating and PR patterns, electrical testing (including capacitance and dielectric loss factor) of an EFD cell with engineered substrate processed by the screen printing method has been made, and data is shown in Table 1. An EFD cell with substrates engineered by spin coating method was also tested for comparison. The comparison sample is shown in Figure 6b,d. Electric testing was done on the 50.7 mm × 66.0 mm sized cell (Figure 6d).

The testing data of capacitance per cell matches the calculated data very well using the equations:

(4) $$1/C_{FP/Oil/Water}=1/C_{FP}+1/C_{oil}$$

(5) $$C=\varepsilon_{0}\varepsilon_{r}A/d$$

where *C_FP/Oil/Water_* is the capacitance of the FP and oil films in water, *C_FP_* is the capacitance of the FP film, *C_oil_* is the capacitance of the oil film, *ε*_0_ is permittivity of vacuum (8.854 × 10^−12^ F/m) , *ε_r_* is permittivity (*ε*_FP_ = 1.93, *ε*_oil_ = 2.2), A is the capacitive area, and *d* is the film thickness. For the printed sample (Figure 6c), the thickness of FP is ~0.6 μm and the thickness of the oil is ~4 μm, so the calculated C is ~37.6 nF, which matches very well with the measured value of 36.3 nF. For the spin-coated sample (Figure 6d), the thickness of FP is ~0.75 μm and the thickness of the oil is ~4 μm, so the calculated C is ~13.2 nF, which also matches well with the measured value of 12.6 nF. For printed samples, the dielectric loss factor per mm^2^ was 7.3 × 10^−^^6^, which was lower than that for spin-coated samples (12 × 10^−^^6^). This confirms that the dielectric quality of screen-printed FP coatings is very good and that screen printing can yield pin-hole free films.

### 3.2. Optical Performance of EFD Devices

**Figure 7:**
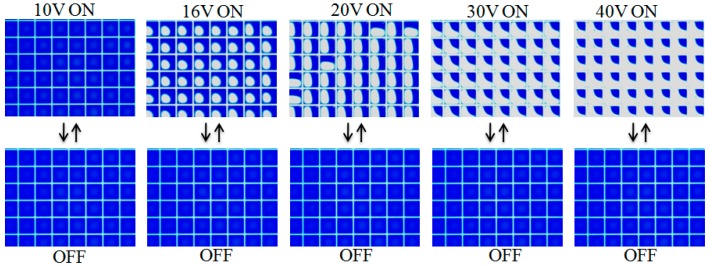
Microscope images of oil movement in each pixel under applied voltage (from 0 V to 40 V). Sixteen volts was needed to activate all the pixels, 20 V to achieve ~50% open area, and 30 V to drive the colored oil to the pixel corners.

EFD devices were fabricated with the engineered substrates as shown in Figure 3, the pixel size was 200 μm × 200 μm, with pixel wall width of 15 μm and height of 6–7 μm. The patterned ITO with “notch” design (shown in Figure 3c) was used as substrate. The notches are areas where there is no conducting ITO coating on the glass. The purpose of the notches is to improve control of the oil residue location when applying a voltage. In this case, the notch area occupied 30% of the pixel area. Figure 7 shows the device performance under various driving voltages. Arrays of 6 × 8 pixels were observed microscopically. Every image shown in Figure 7 represents a stable state, which means that if we keep the voltages on, the oil shape within each pixel will remain the same. All pixels opened when the driving voltage rose to 16 V, and the open area adopted a circular shape occupying ~40% of the pixel area. When the voltage increased to 20 V, the open area became more elliptical in shape and occupied ~60% of the pixel area. All the oil was driven to the pixel corner when the voltage reached 30 V. As voltage is applied to the hydrophobic insulator layer, the decreased interfacial tension between water and insulator will lead to the water contact angle decreasing—the FP surface becomes more hydrophilic, attracting water and repelling the oil. The crystalline and relatively hydrophilic nature of the pixel walls provides strong pinning of the water/oil interface, preventing oil overflow to adjacent pixels.

**Figure 8:**
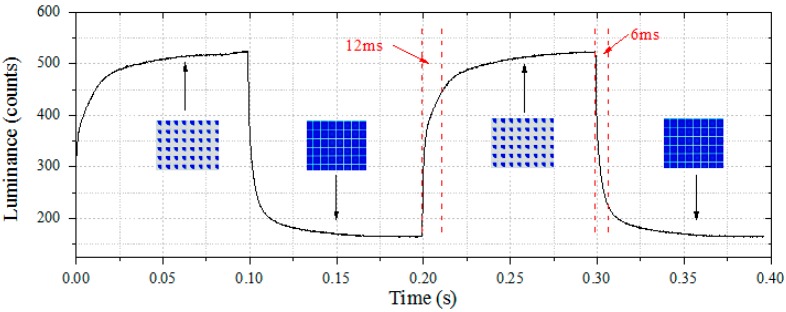
Optical response of EFD measured by a colorimeter. Forty volts of DC voltage was applied at a duty cycle of 50% and 5 Hz frequency (100 ms on/100 ms off). Insets show the optical microscope images of “On” and “Off” states. In this case, the “on” switching time is about 12 ms, and the “off” switching time is 6 ms (corresponding to 80% of maximum change).

Figure 8 shows the optical response of the EFD device measured by a colorimeter driven by a rectangular electrical waveform. The rectangular waveform was generated by a waveform generator and an amplifier; 40 V DC voltage was applied at the duty cycle of 50% and 5 Hz frequency. The incident light was at an angle of 45°, and a detector with area ~1 cm^2^ was positioned normal to the device area. The insets show the optical images of pixels in the Off (0 V) and On (40 V) states. When the device was switched to the “on” state, higher luminance was measured, and vice versa when the device was switched “off”. The highest luminance was ~523 units, and the lowest was ~164 units, which means the highest contrast ratio that could be reached was ~3.2. The transparent pixel walls led to significant light leakage and reduced contrast. A dark photoresist material can be employed to increase the contrast ratio significantly along with further optimization of the optical architecture.

## 4. Experimental

### 4.1. Materials and Equipment

For materials, commercial indium tin oxide (ITO)-coated glass (1.1 mm thick, 100 Ω/□ resistance) purchased from Guangdong Jimmy Glass Technology Ltd. (Foshan, China) (unpatterned) and Leaguer Optronics Co., Ltd. (Shenzhen, China) (patterned) were used as substrates. Amorphous fluoropolymers Teflon AF1600 and AF1600X were purchased from Dupont (Wilmington, DE, USA), and more recently from Chemours Chemical Co., Ltd. (Shanghai, China). The photoresist was a negative type co-developed with a local material supplier. It can be developed in an aqueous solution (KOH) to facilitate industrialization. For equipment, the G2.5 pilot manufacturing line was designed in conjunction with TTMS with individual equipment items manufactured by the followed companies. The cleaning line, screen-printer, hot plates, ovens, and developing line were supplied by Autech (Shenzhen, China), the slit coater by TTMS (Chiba, Japan), and the exposure equipment (PA-4050-5K) by Seiwa (Tokyo, Japan). The reactive ion etching (RIE) tool was supplied by the Institute of Microelectronics, Chinese Academy of Science (Beijing, China), based on our requirements. The equipment was housed in a Class 10,000 environment with the internal process areas maintained at Class 100. All glass was handled in cassettes and via conveyors and robots during processing.

### 4.2. Fabrication Process

**Figure 9:**
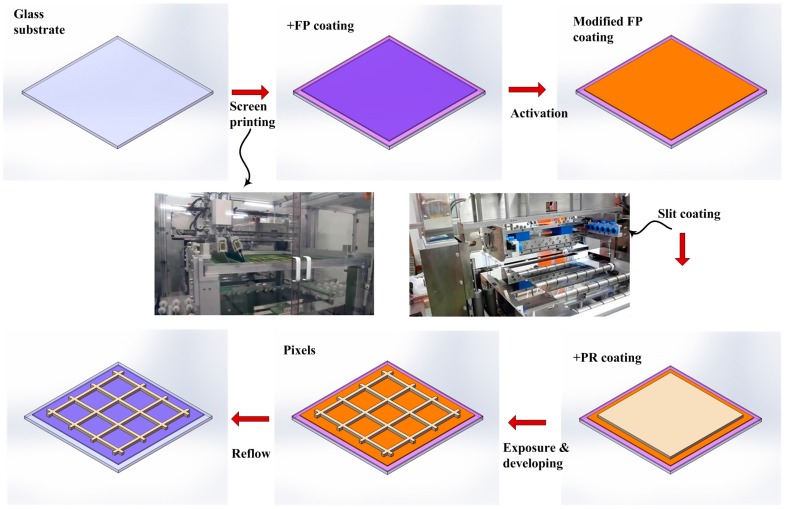
Schematic process flow of fabrication of the large area electrofluidic arrays.

The electrofluidic array fabrication process is illustrated in Figure 9. Commercial ITO substrates are cleaned in an LCD cleaning line for G2.5 glass (400 mm × 500 mm). Amorphous fluoropolymer (FP) layers were coated by screen printer with a thickness in the range 600–1000 nm, followed by baking on hotplates at 100 °C to remove the bulk of fluorocarbon solvent. Heating in an oven at a temperature of 185 °C for 30 min ensured that all solvent was removed. The hydrophobic fluoropolymer surface was then treated by a reactive ion etching process to facilitate its subsequent coating by photoresist. The photoresist was coated onto the modified fluoropolymer surface via slit coating. Thickness of photoresist was uniform (+/− 2%) in the range 5–8 μm. A conventional lithography process was implemented to fabricate the photoresist arrays into pixel walls (or barriers) in an EFD device. A thermal reflow process with a temperature of ~200 °C for 2 h was used to return the surface of the fluoropolymer back to its native hydrophobic state.

### 4.3. Characterisation

The thickness of films was measured via stylus profiler (Dektak XT, BRUKER, Billerica, MA, USA). An impedance analyzer (WAYNE KERR 6500, Chichester, UK) was used to drive and measure the device electrical properties. A waveform generator (Agilent 33500B Series, Santa Clara, CA, USA) and an amplifier (Agilent 35502A) were used to provide the square wave signal with specific voltage amplitude to drive the device. An optical colorimeter (Arges 45, Admesy, Ittervoort, The Netherlands) was used to obtain the optical response of the device as a function of time.

## 5. Conclusions

Large area electrofluidic arrays with 400 mm × 500 mm ITO/glass substrates were successfully fabricated on an automatic production line. Screen printing was employed for efficient coating and patterning of fluoropolymer insulator layers. High coating homogeneity within 3% thickness deviation and high material utilization over 80% were achieved. Slit-coating combined with a standard lithography process were used to fabricate photoresist patterns on the fluoropolymer surface after reactive ion etching. Homogeneous films of both fluoropolymer and photoresist were obtained with a high coating and patterning speed of 5 s and less than 20 s per plate, respectively. The electrofluidic array substrates were evaluated in EFD devices to validate their performance and practicability. Good switching reversibility and speed were achieved with response times of 6–12 ms under a driving voltage of 40 V (threshold voltage 16 V).

## Figures and Tables

**Table 1:** A comparison of electrical characterization of EFD cells with engineered substrates processed by printing process and spin coating process.

Test Information	Large Area Printing Process	6 Inch Spin Coating Process
Cell size	95.2 mm × 95.2 mm	66.0 mm × 50.7 mm
Capacitance per cell	36.3 nF	12.6 nF
Dielectric loss factor per cell	0.066	0.040
Capacitance per mm^2^	4.0 × 10^−3^ nF/mm^2^	3.8 × 10^3^ nF/mm^2^
Dielectric loss factor per mm^2^	7.3 × 10^−6^/mm^2^	12 × 10^−6^/mm^2^

## Supplementary Materials

The following are available online at www.mdpi.com/1996-1944/9/8/707/s1. Figure S1: (a) Photograph of screen with 36 pads of 30 mm × 60 mm. Picture inset is the optical microscope photograph of mesh structure; (b) Amorphous fluoropolymer thickness data measured by stylus profiler on a 400 mm × 500 mm ITO glass substrate; Figure S2: Photoresist thickness data measured by stylus profiler on a 400 mm × 500 mm ITO glass substrate.

## Abbreviations

The following abbreviations are used in this manuscript:

EFD	electrofluidic display
LCD	liquid crystal display
ITO	indium tin oxide
