# Supplementary material for: Coating and Patterning Functional Materials for Large Area Electrofluidic Arrays
Source: Materials (Basel). 2016 Aug 19;9(8):707. doi: 10.3390/ma9080707 (PMC5512529; doi:10.3390/ma9080707)
Supplement: Supplementary file 1 [file materials-09-00707-s001.pdf]

Supplementary Materials: Coating and Patterning Functional Materials for Large Area Electrofluidic Arrays

Hao Wu, Biao Tang, Robert A. Hayes, Yingying Dou, Yuanyuan Guo, Hongwei Jiang and Guofu Zhou

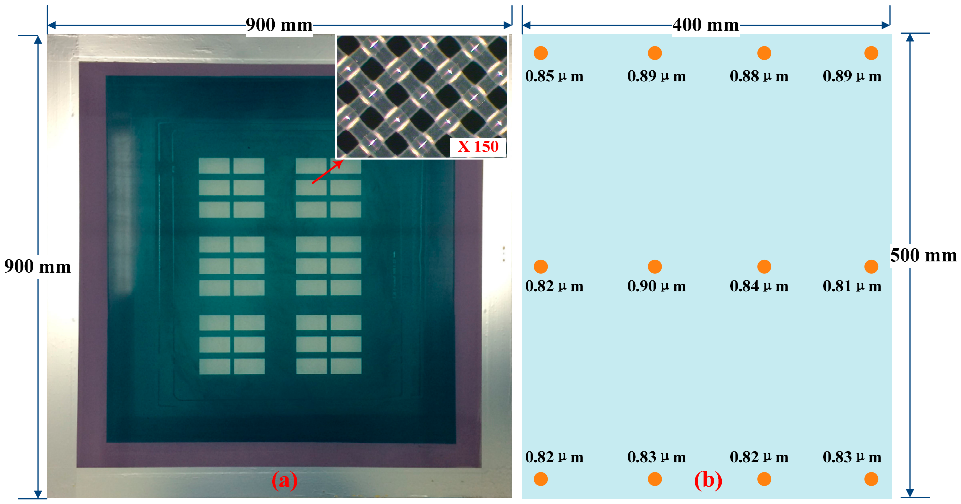

Figure S1. (**a**) Photograph of screen with 36 pads of 30 mm × 60 mm. Picture inset is the optical microscope photograph of mesh structure; (**b**) Amorphous fluoropolymer thickness data measured by stylus profiler on a 400 mm × 500 mm ITO glass substrate.

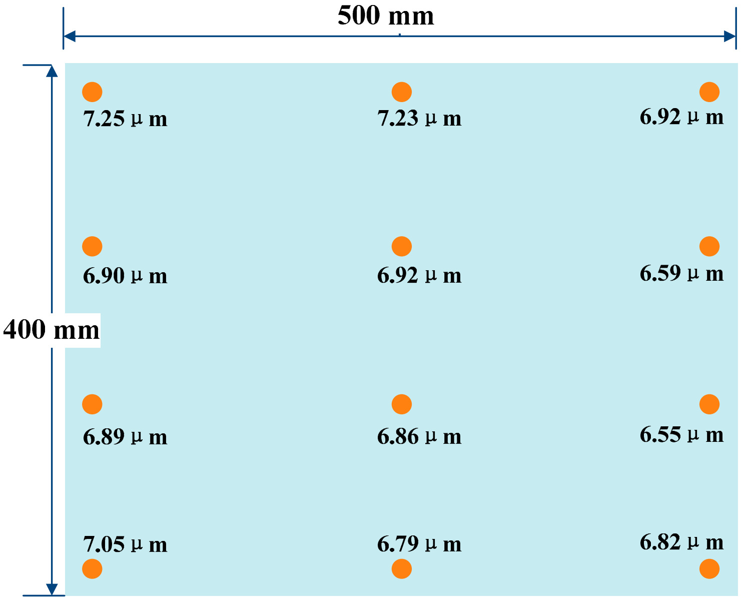

Figure S2. Photoresist thickness data measured by stylus profiler on a 400 mm × 500 mm ITO glass substrate.
